# Line‐Field Confocal Optical Coherence Tomography (LC‐OCT) of Pilomatricoma

**DOI:** 10.1111/pde.70244

**Published:** 2026-05-10

**Authors:** Gerardo Palmisano, Simone Cappilli, Daniele Omar Traini, Enrico Bocchino, Lyna Mtimet, Lucas Boussingault, Mariano Suppa, Ketty Peris, Alessandro Di Stefani

**Affiliations:** ^1^ Dermatologia, Dipartimento di Medicina e Chirurgia Traslazionale Università Cattolica del Sacro Cuore Rome Italy; ^2^ UOC di Dermatologia, Dipartimento Scienze Mediche e Chirurgiche Fondazione Policlinico Universitario A. Gemelli – IRCCS Rome Italy; ^3^ Department of Dermatology Hôpital Erasme, HUB, Université Libre de Bruxelles Brussels Belgium; ^4^ Department of Dermatology Institut Jules Bordet, HUB, Université Libre de Bruxelles, HUB Brussels Belgium; ^5^ Groupe d'Imagerie Cutanée Non Invasive (GICNI) of the Société Française de Dermatologie (SFD) Paris France; ^6^ General Pathology Unit, Department of Woman and Child's Health and Public Health Sciences Fondazione Policlinico Universitario A. Gemelli – IRCCS Rome Italy

## Abstract

Pilomatricoma is a benign cutaneous tumor of hair matrix origin, often presenting in children as a slow‐growing, firm, colored nodule. Line‐field confocal optical coherence tomography (LC‐OCT) is a novel imaging method that enables non‐invasive, quasi‐histologic visualization of the skin. We report the case of a 12‐year‐old patient in whom LC‐OCT allowed the in vivo identification of histological features of pilomatricoma.

1

Pilomatricoma, also known as calcifying epithelioma of Malherbe, is a benign cutaneous tumor originating from immature hair matrix cells. This tumor typically develops mainly on the upper body in the first two decades of life, clinically appearing as a solitary, firm, slow‐growing nodule with one or more colors, ranging from blue‐gray to red to yellow/white [1, 2, 3]. Dermoscopy may be useful to improve the detection of pilomatricoma, providing clues for its identification: reddish homogeneous areas, linear irregular unfocused vessels, yellow‐white structureless areas and structureless blue‐gray areas. However, in approximately 20% of cases histological examination remains mandatory to rule out other skin tumors [1].

Histopathologically, pilomatricoma is characterized by a well‐circumscribed dermal or subcutaneous nodule composed of two main cell populations: basophilic basaloid cells at the periphery and eosinophilic anucleate “shadow” or “ghost” cells at the center [2]. Progressive calcification, ossification, and foreign‐body giant cell reaction are frequently observed, reflecting different stages of tumor evolution [2].

Line‐field confocal optical coherence tomography (LC‐OCT) (DAMAE Medical, Paris, France) is an innovative technique that combines the high‐resolution capabilities of reflectance confocal microscopy (RCM) with the depth‐penetration capabilities of conventional optical coherence tomography (OCT), enabling real‐time imaging of the skin at a quasi‐histologic resolution [4]. The different skin structures can be visualized in vertical and horizontal mode up to a depth of 500 μm, usually including the analysis of the epidermis and the superficial‐mid part of the dermis. Three‐dimensional cubes can also be performed thanks to an integrated software. In recent years, LC‐OCT proved to increase diagnostic accuracy for epithelial skin cancers [5].

Herein we describe a 12‐year‐old boy who was examined for a bluish, asymptomatic, 1.2 × 0.7 mm firm nodule on the left cheek, which developed over the last year (Figure 1a). Structureless gray‐bluish areas were observed as unspecific findings in dermoscopy (Figure 1b). Based on clinical and dermoscopic criteria, the differential diagnosis included blue nevus and pilomatricoma. LC‐OCT sections revealed a regular epidermis overlying dark dermal lobules composed of peripheral bright monomorphic cells encircling a central “shadow” (Figure 2a). The nodule was then surgically excised and histopathological examination confirmed the diagnosis of pilomatricoma, revealing a dermal solid tumor composed of basaloid cells at the periphery and shadow eosinophilic “ghost cells” towards the center, with several areas of calcification, perfectly corresponding to in vivo findings (Figure 2b).

**FIGURE 1:**
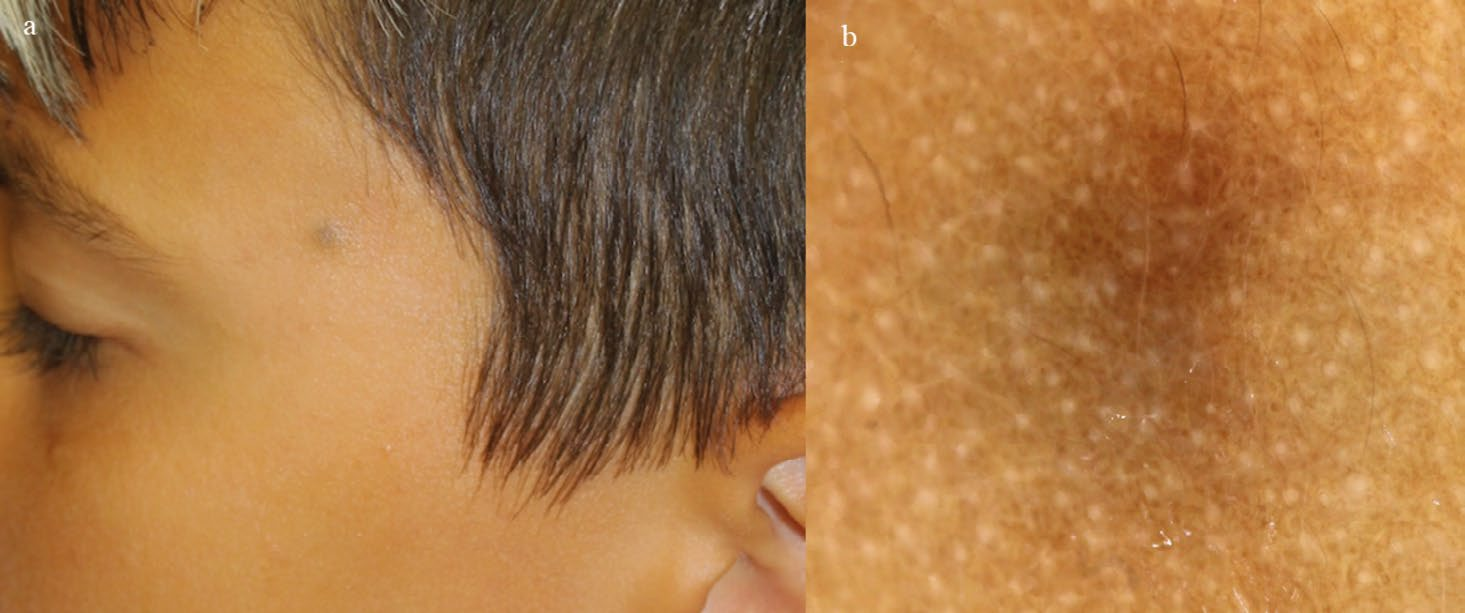
Clinical aspect (a) blue, deep nodule on the left cheek, showing gray‐blue structures zone in dermoscopy (b).

**FIGURE 2:**
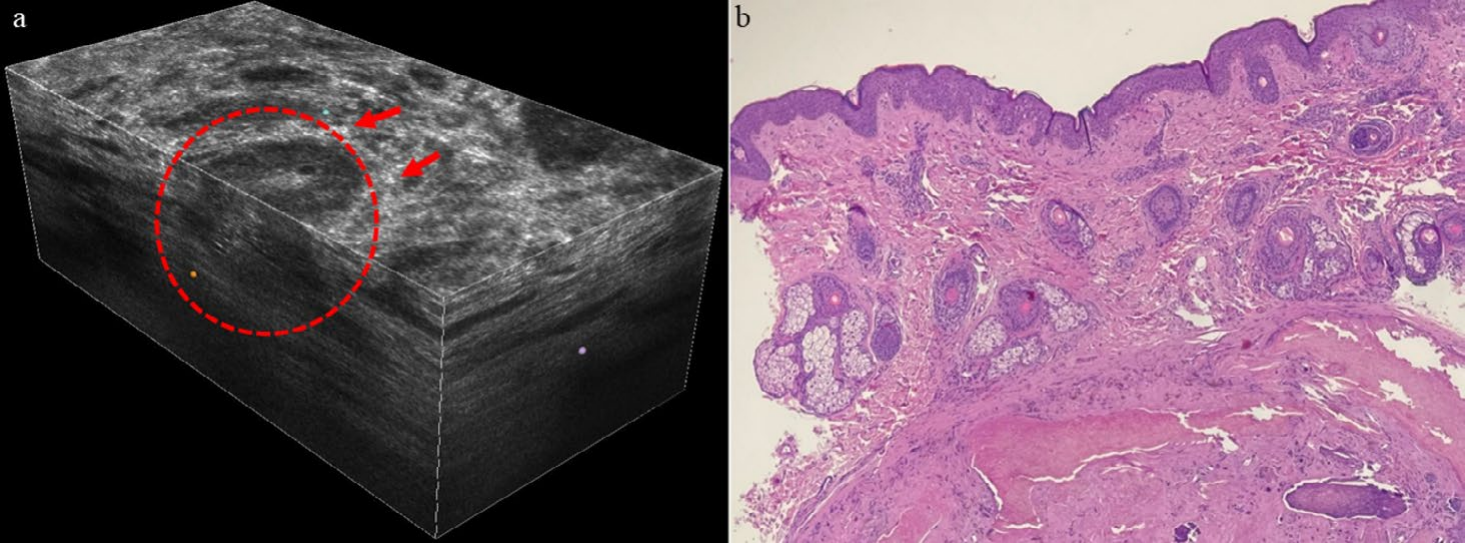
(a) 3D LC‐OCT revealing bright cells (red arrows) surrounding dark circular areas (red circle) in the superficial dermis. (b) Histopathologic examination of the nodule (H&E) revealed a core region of calcification surrounded by basophilic cells on the exterior and squamous cells with a ghost‐like appearance in the middle.

Pilomatricoma may present with a varied morphology, since lesions can be ruptured or non‐ruptured, and may exhibit a different combination of colors, from flesh to bluish, making clinical diagnosis challenging. In the largest case series, multiple irregular whitish structures and streaks were the main dermoscopic features, although dermoscopy of this condition in the pediatric age group is poorly reported in the literature [1, 2, 3]. Ultrasound has been proposed as an additional modality for the diagnosis of pilomatricoma; characteristic findings include well‐defined, ovoid, hypoechoic, heterogeneous masses with a hypoechoic rim, internal echogenic foci, and posterior shadowing at the dermal‐subcutaneous fat junction [2].

Although RCM provides excellent cellular detail, its shallow imaging depth limits its applicability in mainly dermal neoplasms such as pilomatricoma. High‐frequency ultrasound ensures deeper penetration and is widely used in this setting, yet it lacks the resolution required to assess cellular and structural features. LC‐OCT bridges this gap, combining enhanced penetration compared with RCM and superior resolution relative to ultrasound; this distinction should be clearly emphasized.

The application of LC‐OCT allowed the visualization of specific morphological features of pilomatricoma as a lobular architecture with calcific core and bright peripheral ghost cells. These structures correspond to the calcified areas, surrounded by ghost‐like cells and with peripheral basophilic cells as observed in the histopathology exam [2]. Nevertheless, the deep dermal location of this lesion could exceed the limit of LC‐OCT (500 𝜇m).

Our case report suggests that LC‐OCT could represent a promising advancement in the non‐invasive diagnosis of pilomatricoma, offering high‐resolution imaging that improves diagnostic accuracy and patient care. Larger case series and comparative imaging studies will be necessary to define the diagnostic role of LC‐OCT in this context.

## Consent

All patients in this manuscript have given written informed consent for participation in the study and the use of their de‐identified, anonymized, aggregated data and their case details (including photographs) for publication.

## Conflicts of Interest

The authors declare no conflicts of interest.

## Data Availability

The data that support the findings of this study are available on request from the corresponding author. The data are not publicly available due to privacy or ethical restrictions.
